# History of a Case of Tetanus

**Published:** 1903-02

**Authors:** R. Barnes

**Affiliations:** London, Ont.


### HISTORY OF A CASE OF TETANUS.

BY R. BARNES, V.S.,
LONDON, ONT.

ON October 16, 1902, I was called to see a bay horse, ten years old, weight about 1100 pounds, used as a cab horse, which presented the following symptoms: cervical and muscles of back rigid, ears pricked, head poked out, tail slightly elevated, difficult respiration; upon the least excitement he appeared very nervous, would retract eye until membrana nictitans would obscure it; temperature 103°, pulse 80; could masticate very little. Diagnosed it tetanus. Owner informed me it had been acting strange for a few days; I examined it to find if possible the point of infection, but was unable to do so.

Decided to treat it. Had the animal at once removed to a darkened, airy stall, and placed in slings; ordered but one attendant to look after him; his food was to be of a sloppy character and abundance of fresh water to be kept so he could get it; ordered enemas three or four times a day. Gave potass. nitras, 1 drachm; mag. sulph., 1 ounce; sod. hypo. sulph., ½ ounce, to be repeated three times daily as long as he was able to take it in his food.

Left solution as follows: Acid, carbolic, 2 drachms; glycerin, 2 drachms; aquæ dest., add 4 ounces; 1 drachm to be given hypodermically every two hours.

Second day, not much change except in pulse, which had fallen to 60.

Third, fourth, and fifth days, scarcely any change except in pulse, which had fallen to 35; stopped enemas, as bowels were moving freely.

Sixth and seventh days, not much change, pulse down to 30; ordered hypodermics every three hours.

Eighth, ninth, tenth, and eleventh days, unfavorable symptoms gradually increasing. On twelfth day could not drink, extremely nervous and excitable, and no doubt would have fallen if not protected by slings; pulse 90; ordered hypodermic every two hours and enemas as before.

The thirteenth, fourteenth, and fifteenth days, symptoms abating; on sixteenth day was able to suck down some gruel; ordered same powder to be given in feed as usual; pulse 45.

The seventeenth, eighteenth, and nineteenth days, improved each day; ordered hypodermic every three hours; pulse 35.

The twentieth, twenty-first, and twenty-second days, symptoms more favorable, could masticate slowly, choice bits of clover hay were given; hypodermics every six hours.

The twenty-third, twenty-fourth, and twenty-fifth days, doing remarkably well, eating well; hypodermics three times a day.

The twenty-sixth, twenty-seventh, and twenty-eighth days, rigidity of muscles disappearing; stopped hypodermics.

The twenty-ninth and thirtieth days, continued to improve; removed slings.

The thirty-second day, ceased giving medicines; could walk around stall nicely. Ordered that light be gradually admitted into stall. By the fortieth day symptoms entirely disappeared. Ordered slow walking exercise, which was increased until on the fiftieth day, the horse was at his old occupation, that of pulling a hack.

Owner reports that the only difference he notices is that he is more nervous than previous to his sickness.

